# Successful Conversion Surgery Following S-1 Plus Oxaliplatin Combined with Nivolumab Therapy for a Preoperatively Diagnosed Gastric Adenocarcinoma with Enteroblastic Differentiation: A Case Report

**DOI:** 10.70352/scrj.cr.24-0134

**Published:** 2025-04-09

**Authors:** Mizuki Fukuda, Naoto Takahashi, Yoshitaka Ishikawa, Naoki Toya, Kosuke Sasuga, Shoko Handa, Syun Sato, Fumiaki Yano, Ken Eto

**Affiliations:** 1 Department of Surgery, The Jikei University, Kashiwa Hospital, Kashiwa, Chiba, Japan; 2 Department of Pathology, The Jikei University, Kashiwa Hospital, Kashiwa, Chiba, Japan; 3 Department of Gastrointestinal Surgery, The Jikei University School of Medicine, Tokyo, Japan

**Keywords:** gastric adenocarcinoma with enteroblastic differentiation, Stage IV, peritoneal dissemination, nivolumab, conversion surgery

## Abstract

**INTRODUCTION:**

Gastric adenocarcinoma with enteroblastic differentiation (GAED) is a rare subtype of gastric cancer known for its aggressive nature compared to conventional gastric adenocarcinoma. Due to its rarity and high malignancy, reports of successful medication therapy for Stage IV GAED are scarce. In this case, we report a GAED patient with peritoneal dissemination who responded well to combination chemotherapy with nivolumab, leading to the possibility of conversion surgery.

**CASE PRESENTATION:**

A 74-year-old man presented with anemia and was diagnosed with GAED involving pancreatic infiltration and peritoneal dissemination. As first-line treatment, he underwent 9 cycles of S-1 and oxaliplatin chemotherapy combined with nivolumab. The tumor showed remarkable shrinkage. Staging laparoscopy revealed the disappearance of peritoneal nodules, and negative peritoneal cytology was confirmed intraoperatively. Consequently, conversion surgery was performed, involving laparoscopic distal gastrectomy with D2 lymph node dissection and Roux-en-Y reconstruction. Pathological examination showed ypT2N0M0, ypStage IB, with a chemotherapy response graded at 2a. Although peritoneal dissemination recurred 4 months after surgery, restarting nivolumab monotherapy significantly reduced ascites, and the patient maintained a partial response.

**CONCLUSIONS:**

Stage IV GAED is associated with a poor prognosis; however, the advent of immune checkpoint inhibitors has expanded treatment options for these patients. In this case, we propose a personalized treatment strategy for GAED with peritoneal metastases that may improve clinical outcomes.

## Abbreviations

AFP
: alpha-fetoprotein
CA125
: cancer antigen 125
CGA
: conventional gastric adenocarcinoma
CLDN
: claudin
CPS
: combined positive score
CS
: conversion surgery
CT
: computed tomography
CY0
: negative peritoneal cytology
GAED
: gastric adenocarcinoma with enteroblastic differentiation
GPC3
: glypican-3
HER2
: human epidermal growth factor receptor 2
ICIs
: immune checkpoint inhibitors
MP
: muscularis propria
MSI
: microsatellite instability
PD-L1
: programmed cell death ligand 1
PR
: partial response
SALL-4
: spalt-like transcription factor 4
SOX
: S-1 plus oxaliplatin

## INTRODUCTION

Gastric adenocarcinoma with enteroblastic differentiation (GAED) is classified as a type of alpha-fetoprotein (AFP)-producing gastric cancer, and it is categorized as a special type of malignant epithelial tumor in the 15th edition of the Japanese Classification of Gastric Carcinoma.^1)^ GAED shows a histological pattern similar to embryonic gastrointestinal epithelium^2)^ and tends to be more aggressive than conventional gastric adenocarcinoma (CGA), with a higher incidence of vascular invasion and liver metastasis, leading to a poor prognosis.^3,4)^ Although GAED accounts for only 2.2% of all gastric cancers,^5)^ its high malignancy has attracted increasing attention recently, and its pathophysiology is gradually being elucidated.

No specific treatment is established for GAED; the treatment approach is generally similar to CGA. For resectable GAED, curative tumor resection is performed. In cases where cancer is unresectable at initial diagnosis, chemotherapy becomes the primary treatment option. However, no standard chemotherapy regimen has been established specifically for GAED, and it remains unclear whether therapies used for CGA yield equivalent results due to the disease’s rarity. Additionally, because GAED is a disease with a poor prognosis, there have been very few reports of successful pharmacotherapy for Stage IV GAED. Molecular targeted therapies and immune checkpoint inhibitors (ICIs) have effectively treated CGA. Recent studies have reported a higher expression of human epidermal growth factor receptor 2 (HER2) in GAED, suggesting that trastuzumab may be effective in HER2-positive Stage IV GAED.^5–7)^ Furthermore, GAED often expresses high programmed cell death ligand 1 (PD-L1), indicating that ICIs may also be effective.^8)^

In this report, we describe a case of Stage IV GAED with peritoneal dissemination treated with a first-line regimen of S-1 plus oxaliplatin (SOX) combined with nivolumab. The tumor showed remarkable shrinkage, and the peritoneal dissemination disappeared, enabling conversion surgery (CS). This case highlights the potential for ICIs to be effective in treating advanced GAED, especially in combination with other chemotherapy regimens, leading to favorable outcomes even in cases of Stage IV GAED.

## CASE PRESENTATION

**Fig. 1:**
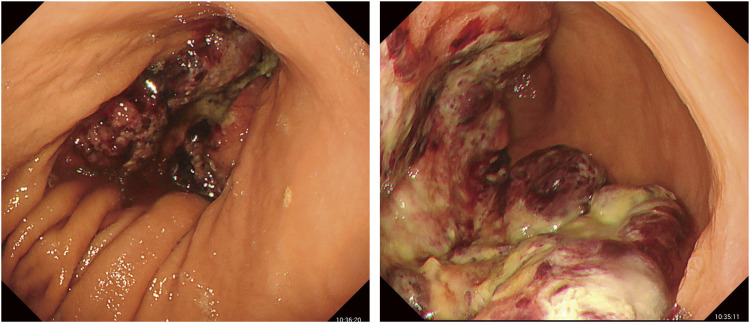
Esophagogastroduodenoscopy. A 50-mm Type 3 tumor was observed on the greater curvature of the lower body of the stomach.

**Fig. 2:**
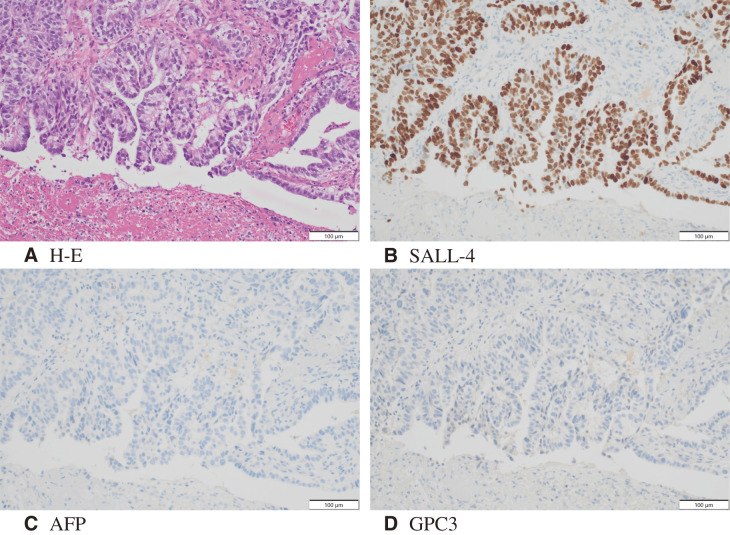
Pathological findings of the biopsy specimen. (**A**) The tumor cells, characterized by a clear cytoplasm, proliferated in glandular and papillary patterns. (**B**) Additional immunohistochemistry shows that the cells were positive for the fetal tumor protein SALL-4, leading to a diagnosis of GAED. (**C**) AFP was negative. (**D**) GPC3 was negative. AFP, alpha-fetoprotein; GAED, gastric adenocarcinoma with enteroblastic differentiation; GPC3, glypican-3; H-E, hematoxylin and eosin; SALL-4, spalt-like transcription factor 4

**Fig. 3:**
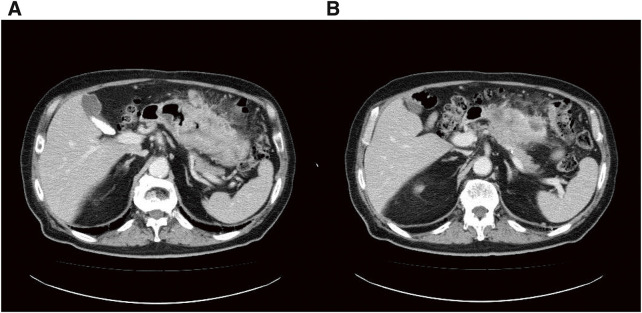
Contrast-enhanced CT findings. The contrast-enhanced CT showed wall thickening in the body of the stomach, as well as (**A**) peritoneal disseminated nodules and (**B**) pancreatic infiltration. CT, computed tomography

**Fig. 4:**
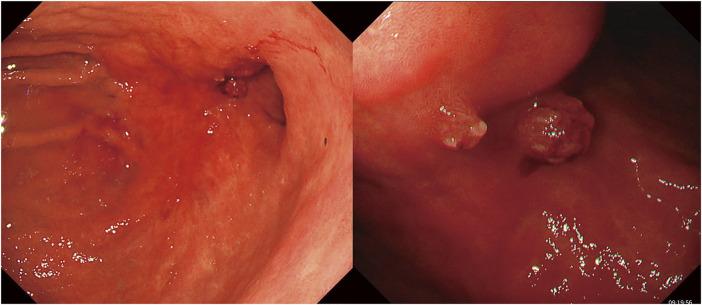
Esophagogastroduodenoscopy after nivolumab combination chemotherapy. The tumor in the greater curvature of the lower body of the stomach had significantly shrunk.

**Fig. 5:**
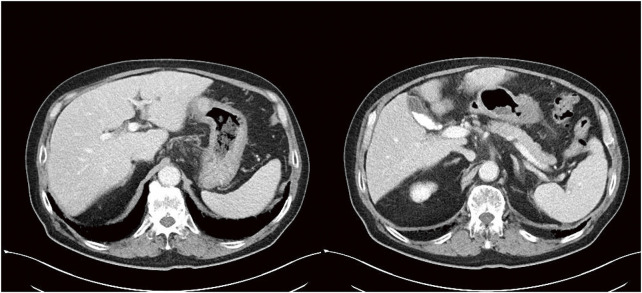
Contrast-enhanced CT findings after nivolumab combination chemotherapy. The stomach wall thickening had decreased, and the findings of peritoneal disseminated nodules and pancreatic infiltration had disappeared. CT, computed tomography

**Fig. 6:**
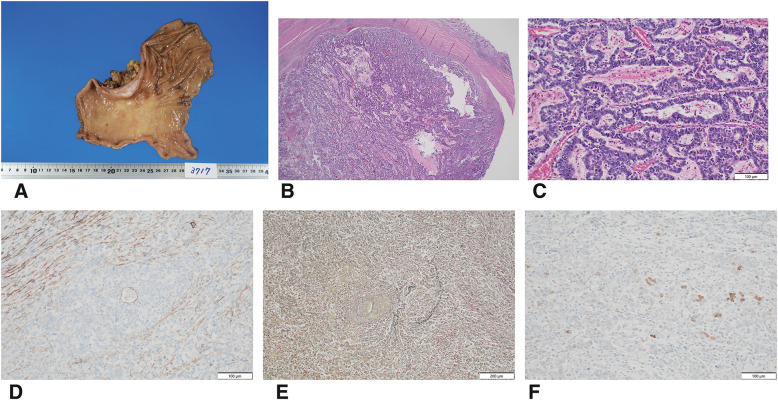
Pathological findings. (**A**) Macroscopic findings: a Type 5 tumor measuring 44 × 30 mm on the lower body and greater curvature of the stomach was observed. (**B**) In the microscopic image, no tumors were observed within the mucosa, but a papillary proliferative tumor was noted spreading in the submucosal layer. (**C**) High magnification image of (**B**). (**D**) D2-40 staining revealed lymphatic invasion. (**E**) EVG staining revealed venous invasion. (**F**) The tumor had degenerated due to nivolumab combination chemotherapy, but the majority was SALL-4 positive in immunohistochemical staining. EVG, elastica van Gieson; SALL-4, spalt-like transcription factor 4

**Fig. 7:**
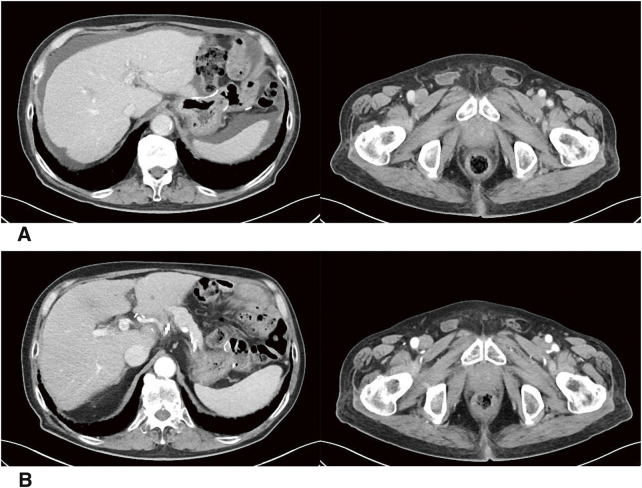
Postoperative CT findings. Four months after surgery, no evident peritoneal dissemination nodules were observed; however, ascites were detected, raising suspicion of recurrent peritoneal dissemination (**A**). Nivolumab monotherapy was resumed, and the ascites gradually decreased. The patient has continued to show PR on CT scans after 17 courses of nivolumab (**B**). CT, computed tomography; PR, partial response

**Fig. 8:**
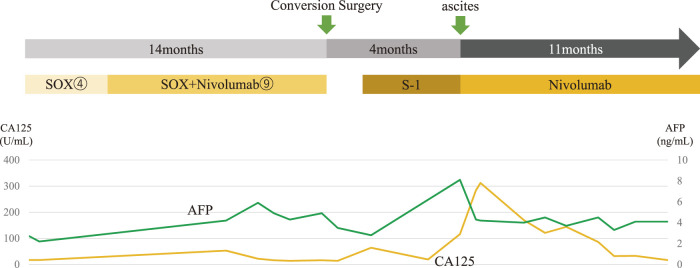
Summary of the course of this case. After administering 9 courses of SOX plus nivolumab therapy following the SOX regimen, tumor reduction effects and the disappearance of peritoneal metastatic nodules were confirmed, leading to a diagnostic laparoscopy, which made conversion surgery possible. Although S-1 was given as adjuvant therapy after surgery, the patient had bilateral inguinal hernias 4 months after surgery, and contrast-enhanced CT revealed the presence of ascites. Tumor markers were also elevated, prompting the resumption of nivolumab monotherapy for suspected recurrence of peritoneal dissemination. Subsequently, the ascites decreased, and tumor markers gradually normalized. Twenty-nine months have passed since the initial consultation, and tumor control remains good. CT, computed tomography; SOX, S-1 plus oxaliplatin

A 74-year-old male visited a hospital complaining of fatigue and was found to have anemia with a hemoglobin level of 5.0 g/dL. He was referred to our hospital for further evaluation and treatment. His past medical history included postoperative abdominal aortic aneurysm, angina pectoris, hypertension, and diabetes mellitus. Upper gastrointestinal endoscopy (**Fig. 1**) revealed a 50-mm Type 3 tumor in the greater curvature of the lower body of the stomach. Biopsy results confirmed the presence of adenocarcinoma. Examination of the biopsied tissue revealed tumor cells with clear cytoplasm proliferating in a papillary pattern, which warranted further immunohistochemical analysis. The immunohistochemical results were positive for spalt-like transcription factor 4 (SALL-4) and negative for both AFP and glypican-3 (GPC3) (**Fig. 2**), which facilitated a diagnosis of GAED. As shown in **Table 1**, blood tests revealed no elevation of tumor markers, including AFP. Contrast-enhanced computed tomography (CT) (**Fig. 3**) revealed gastric wall thickening, suspected pancreatic invasion, and peritoneal dissemination nodules. The patient was diagnosed with unresectable advanced gastric cancer. During SOX therapy, molecular markers of this tumor were determined to be negative for HER2, negative for claudin (CLDN) 18.2, with a combined positive score (CPS) of >5, and high microsatellite instability (MSI). Based on these findings, the introduction of ICIs was deemed desirable, and SOX plus nivolumab was initiated. After 7 courses of SOX plus nivolumab, upper gastrointestinal endoscopy (**Fig. 4**) showed marked tumor shrinkage. After 9 courses, contrast-enhanced CT (**Fig. 5**) confirmed the reduction of the primary lesion and the disappearance of the peritoneal dissemination nodules. One year and two months after the initial diagnosis, staging laparoscopy was performed, revealing no evidence of peritoneal dissemination or metastasis, and negative peritoneal cytology (CY0) was confirmed intraoperatively. The posterior wall of the stomach showed adhesion to the pancreas, making dissection difficult, but no apparent pancreatic invasion was observed. CS was considered feasible, and laparoscopic distal gastrectomy with D2 lymph node dissection and Roux-en-Y reconstruction was performed. The patient developed a postoperative pancreatic fistula, but it improved with conservative treatment, and he was discharged on postoperative day 36. Pathological examination (**Fig. 6**) revealed a Type 5 tumor measuring 44 × 30 mm in the anterior wall to the greater curvature of the middle and lower stomach, with negative resection margins. Tumor cells were distributed in the submucosal tissue or more profoundly, but no cancer cells were found in the superficial mucosa. Although the tumor cells were degenerated because of pharmacotherapy, most were SALL-4-positive, consistent with GAED. The depth of invasion was T2 (muscularis propria [MP]). Vascular invasion and lymphatic invasion were observed. A total of 14 lymph nodes were dissected, but no metastasis was observed, and there were no scars from metastasis that had disappeared with pharmacotherapy. The final pathological diagnosis was gastric cancer of the middle and lower stomach, anterior wall to greater curvature, Type 5, GAED, ypT2(MP), INFa, Ly1a, V1a, pPM0, pDM0, ypN0, ycM0(H0, P0, CY0), and ypStage IB. The effect of chemotherapy was classified as Grade 2a. Following surgery, adjuvant chemotherapy with S-1 monotherapy was initiated; however, 4 months postoperatively, bilateral inguinal hernias were identified. An abdominal CT scan (**Fig. 7A**) revealed no peritoneal dissemination nodules, although ascites were present. Cytological analysis of the ascitic fluid classified it as Class II, with no other identifiable causes for the ascites. Additionally, elevated cancer antigen 125 (CA125) levels suggested a potential recurrence of peritoneal dissemination. Treatment with nivolumab monotherapy was subsequently resumed. After 17 cycles, contrast-enhanced CT (**Fig. 7B**) indicated a reduction in ascites, and the symptoms associated with the inguinal hernias had resolved. CA125 levels also showed a trend toward normalization, and the patient was evaluated as having achieved a partial response (PR). **Figure 8** summarizes the clinical course of this case. The patient has continued nivolumab monotherapy and has currently achieved a survival period of 29 months since the initial diagnosis.

**Table 1:** Blood test findings

WBC	10200/μL	AST	7 U/L	Na	138 mmol/L
RBC	259 × 10^4^/μL	ALT	6 U/L	K	4.4 mmol/L
Hb	5.0 g/dL	LDH	122 U/L	Cl	107 mmol/L
Ht	19.4%	ChE	107 U/L	Ca	7.9 mg/dL
MCV	74.9 fL	T-Bil	0.6 mg/dL	CRP	1.17 mg/dL
MCH	19.3 pg	ALP	83 U/L	CEA	2.8 ng/mL
MCHC	25.8 g/dL	γ-GTP	14 U/L	CA19-9	21 U/mL
Plt	35.3 × 10^4^/μL	TP	5.3 g/dL	AFP	2.7 ng/mL
		Alb	2.6 g/dL	CA125	17 U/mL
		UN	10 mg/dL		
		Cr	0.52 mg/dL		

*γ*-GTP, gamma-glutamyl transferase; AFP, alpha-fetoprotein; Alb, albumin; ALP, alkaline phosphatase; ALT, alanine aminotransferase; AST, aspartate aminotransferase; CA19-9, carbohydrate antigen 19-9; CA125, cancer antigen 125; CEA, carcinoembryonic antigen; ChE, cholinesterase; Cr, creatinine; CRP, C-reactive protein; Hb, hemoglobin; Ht, hematocrit; LDH, lactate dehydrogenase; MCH, mean corpuscular hemoglobin; MCHC, mean corpuscular hemoglobin concentration; MCV, mean corpuscular volume; Plt, platelet; RBC, red blood cell; T-Bil, total bilirubin; TP, total protein; UN, urea nitrogen; WBC, white blood cell

## DISCUSSION

We have encountered a case of GAED, recognized as a distinct subtype in the Japanese Classification of Gastric Carcinoma.^1)^ In this case report, we analyze the preoperative diagnostic process and highlight that the patient successfully underwent CS following chemotherapy, which included nivolumab, despite being classified as Stage IV with peritoneal metastases.

GAED was first reported by Matsunou et al. in 1994.^2)^ Histologically, it is characterized by (i) columnar cancer cells growing in a tubular or papillary pattern; (ii) cancer cells with clear cytoplasm and oval nuclei located basally; and (iii) clear cytoplasm rich in glycogen granules without mucin.^2)^ The diagnosis of GAED was made after immunohistochemistry confirmed positivity for fetal tumor proteins such as AFP, GPC-3, or SALL-4.^9,10)^ In our case, a pathological biopsy examination revealed tumor cells with clear cytoplasm proliferating in a papillary pattern, which tested positive for SALL-4 in immunohistochemistry, leading to the diagnosis of GAED. We would like to emphasize that GAED was diagnosed solely based on the initial biopsy conducted before surgery in this case. Because GAED typically coexists with CGA, CGA often covers the tumor surface while GAED invades more profoundly into the submucosa.^3,11)^ Since endoscopic biopsies primarily sample the tumor surface, many cases are initially diagnosed as CGA, with GAED being identified only after surgical resection.^12,13)^

Clinically, the issue with GAED is its poor prognosis compared to CGA, as it has a higher incidence of vascular invasion and liver metastasis.^3,4)^ Compared to CGA, GAED shows higher rates of lymphatic invasion (76% vs. 41%), venous invasion (72% vs. 31%), liver metastasis (31% vs. 6%), and lymph node metastasis (69% vs. 38%). The 1-year survival rate is 38.7%, and even in cases of curative resection, it is reported to be 66.7%.^4)^ In our case, although venous and lymphatic invasion were observed, there was no clear evidence of lymph node or liver metastasis, and peritoneal dissemination was present, making it atypical. The frequency of peritoneal dissemination in GAED does not significantly differ from that in CGA,^7)^ and no studies have yet summarized cases of GAED with peritoneal dissemination. Therefore, although the prognosis remains unclear, the diagnosis of Stage IV GAED led us to anticipate a poor prognosis at the time of diagnosis. Despite the high grade of malignancy, there is no established chemotherapy specific to GAED, and treatment follows CGA guidelines. Wang et al. reported on the molecular characteristics of 37 cases of GAED, noting a higher expression of PD-L1 and frequent cases with CPS >5.^8)^ They concluded that ICIs could be an effective treatment option for GAED. Nivolumab has shown efficacy as a third-line treatment for unresectable or recurrent gastric cancer in the ATTRACTION-2 trial.^14)^ Furthermore, the CheckMate649 trial demonstrated that, as a first-line therapy for previously untreated HER2-negative Stage IV gastric cancer, nivolumab combined with chemotherapy significantly extended overall survival and progression-free survival compared to chemotherapy alone.^15)^ Based on these findings, nivolumab was approved in Japan in 2017, and since November 2021, nivolumab, in combination with chemotherapy, has been recommended as a first-line treatment in the gastric cancer guidelines.^16)^ There have been reports of nivolumab usage in GAED, with cases where it was effective against liver metastases as a third-line treatment,^17)^ as well as cases where it was used as a first-line treatment but failed to control tumor progression.^18)^ To our knowledge, our case is the first reported case where SOX plus nivolumab therapy was successful as a first-line treatment for Stage IV GAED, enabling CS. In our case, introducing ICIs in the first-line treatment was desirable because genetic testing revealed negative for HER2, negative for CLDN 18.2, CPS of >5, and high MSI. The SOX plus nivolumab therapy was effective, allowing us to proceed with CS.

In this case, S-1 monotherapy was used as postoperative adjuvant therapy; however, there are no clear guidelines for adjuvant treatment in GAED. Many cases adhere to the CGA guidelines for postoperative adjuvant therapy.^19)^ While there is no definitive evidence regarding postoperative adjuvant chemotherapy following CS for CGA, S-1 monotherapy or S-1-containing regimens are often selected.^20,21)^ In this case, S-1 monotherapy was chosen.

Postoperatively, ascites suggestive of recurrence were observed, but cytology results indicated Class II. However, tumor markers were elevated, and no other causes of ascites accumulation were identified, suggesting a recurrence of GAED. Based on the genetic test results, we resumed nivolumab monotherapy. Subsequently, the ascites markedly decreased, indicating that nivolumab alone might also be effective against GAED. As a result, the patient has survived 2 years and 5 months since the initial diagnosis.

## CONCLUSIONS

We encountered a Stage IV GAED case that responded well to nivolumab, resulting in improved survival outcomes after CS. As the options for pharmacotherapy expand, there is potential to improve prognosis even in high-grade malignancies like GAED.

## DECLARATIONS

### Funding

We received no financial support from any funding sources.

### Authors’ contributions

All authors have read and approved the manuscript, and they are responsible for the manuscript.

Naoto Takahashi and Yoshitaka Ishikawa were responsible for the patient’s clinical management and data acquisition.

Kosuke Sasuga, Shoko Handa, and Syun Sato provided information on the pathological findings.

Mizuki Fukuda and Naoto Takahashi were responsible for drafting the manuscript and interpreting the data.

Naoki Toya, Fumiaki Yano, and Ken Eto were accountable for the critical revision of the manuscript.

### Availability of data and materials

All data generated or analyzed during this study are included in the published article.

### Ethics approval and consent to participate

This study was carried out using the principles of the Declaration of Helsinki. This work does not require ethical considerations or approval.

### Consent for publication

The patient has consented to publication, including images.

### Competing interests

The authors declare that they have no competing interests.
